# Evaluation of Nurse Navigator Support for Patients During Telehealth Neurosurgery Clinics

**DOI:** 10.1177/23743735241257385

**Published:** 2024-05-31

**Authors:** Giles Critchley, Phylis Harvey, Rose Saunders, Simon John, Keith Todd

**Affiliations:** 1 Department of Neurosurgery, Dunedin School of Medicine, University of Otago, Dunedin, New Zealand; 2 Neurosurgery Navigator Team, Southland Hospital, Kew, Invercargill, New Zealand; 3 Department of Neurosurgery, 67587 Christchurch Hospital, Christchurch, New Zealand; 4 South Island Neurosurgery Services, 67587 Christchurch Hospital, Christchurch, New Zealand

**Keywords:** Patient satisfaction, telehealth clinics, neurosurgery, nurse navigator, service improvement

## Abstract

Telehealth clinics have been used in many specialities, including neurosurgery, to improve access for patients in rural communities. The introduction of nurse navigators involved with the patient before, during and after the clinic was evaluated. Clinics were held in a rural hospital with a nurse navigator present in the clinic with the patient, and the physician consulting remotely. A patient satisfaction survey and audit were conducted following ten telehealth clinics. Twenty-one new patients were able to be contacted out of 31 (68%) with an 11 question structured survey. Eighteen out of 21 (86%) stated they were satisfied with the quality of the clinic compared with an in-person clinic. Overall satisfaction scores of 7–10 were scored by 18/21 patients (86%) on a scale of 1–10. An estimated 10785 km of travel was saved for patients going to a rural hospital clinic rather than the neurosurgical centre. This study shows that the supportive role of nurse navigators throughout the patient telehealth clinic pathway merits further continuing evaluation.

## Introduction

Face-to-face (in-person) clinic appointments with a specialist are being challenged by the use of telehealth clinics. This has particularly increased following the covid-19 pandemic.^1-3^ In the United States in 2021, 37% of adults had used telemedicine in the past 12 months.^2^ Telemedicine has been utilised in neurosurgery^4-6^ as well as other specialities. Many professional organisations and governments have issued guidelines as to the use of telehealth clinics.^3,7^

This study was designed to evaluate the introduction of televideo telehealth clinics in a rural area supported by nurse navigators. These telehealth clinics aimed to reproduce a hospital clinic environment with nurse support for patients with reduced travel, in a local rural hospital setting and meeting the specialist via a video screen rather than in-person.

As a service improvement project 10 telehealth clinics were set up from July to October 2023. These clinics were run by a neurosurgeon based at the Hospital ‘A’ consulting with long-waiting patients who live in the rural district who would usually have travelled to Hospital ‘B’ for their neurosurgery appointment. The appointment of specialist neurosurgical nurse navigators was made to identify and contact these patients and coordinate their clinic appointments. The nurse navigators were present at the clinic appointments with the patient. The nurse navigator role during the clinic appointment was to coordinate the telehealth technology, to be available in person to explain the specialist discussion with the patient and coordinate follow-up in a rural area. The selection of appointments was managed by the rural district hospital-based navigator team located at Hospital ‘C’ who initially selected the patients based on clinical urgency, suitability for telehealth appointment and length of wait.

The clinics took place in three rural hospitals – Hospital ‘C’, Hospital ‘D’ and Hospital ‘E’. The patient travelled as little as possible, the surgeon remained in the specialist hospital and the nurse navigator travelled to the rural site.

This patient satisfaction survey was designed to gauge the priority for patients of their needs when organising a venue for clinics, and the satisfaction of patients attending a neurosurgery First Specialist Appointment (FSA) telehealth clinic with nurse navigator present as opposed to a traditional in-person clinic with the neurosurgeon. Additional audit information was used to determine the financial impact of the telehealth clinics.

## Methods

New (FSA) patients were taken from the outpatient waiting list prioritising the longest waiting patients in a rural catchment area. Patients were selected by the neurosurgery nurse navigator team and also triaged by the specialist physician as to the potential suitability for a telehealth clinic. Pre-clinic imaging including MRI and CT scans were performed when indicated and the images shared with the patient during the appointment.

A total of 10 clinics were provided, with a total of 31 FSA patients and 36 follow-up patients attending. This review of patient satisfaction and audit focused on the 31 FSA patients only.

A Nurse Navigator from Hospital ‘C’ attended the appointments of 26 of the FSA patients. The specialist physician was at the tertiary referral hospital – Hospital ‘A’ (357 km from Hospital ‘B’) and patients attended a clinic with the Nurse Navigator at the appointed regional district hospital site – Hospital ‘C’ – (207 km from Hospital ‘B’, 565 km from Hospital ‘A’), Hospital ‘D’ (214 km from Hospital ‘B’ 442 km from Hospital) or Hospital ‘E’ Hospital (152 km from Hospital ‘B’, 510 km from Hospital ‘A’)

The audio-visual system Microsoft Teams was used. For the initial four clinics the local hospital-based IT system was used. For the following six clinics, a customised laptop with prearranged settings was used to standardise audio-visual quality. The survey was designed to evaluate patient satisfaction with telehealth, and the contribution of the neurosurgical nurse navigator to the efficiency of the clinic and patient satisfaction. A structured questionnaire of 11 questions with the opportunity for additional comments was designed to assess this (Table 1). The performance areas of interest were below: patient satisfaction of attending a telehealth clinic appointment, reduction in mileage from car travel for patients, reduction in time spent travelling by car for patients, and patient outcomes for FSA patients attending telehealth clinics and non-attendance rates.

**Table 1.** Patient Satisfaction Survey.

**Patient Satisfaction Survey** Were you aware this appointment was a virtual/telehealth appointment? Were you happy with this arrangement pre-appointment? Were you well prepared at the beginning of the appointment by the nurse? How would you rate the quality of the audio & visual of the telehealth appointment? Were you able to understand what you were being told by the consultant? Do you rate this as a similar quality meeting with your consultant as an in-person appointment? Did the nurse have to explain anything after the appointment? Would you be happy to have a telehealth appointment again? (or prefer an in-person next time?) Any suggestions on how we can improve this new way meeting with patients? Overall level of satisfaction from the clinic - out of 10 (1 being very unsatisfied, 10 being very satisfied) Would you prefer to see the neurosurgeon in-person next time, or happy with the virtual clinic set-up?

Other relevant comments.

## Results

The median age of the 21 patients surveyed was 64 years old (range 33-84 yrs). There were 9 men and 12 women.

### Patient Satisfaction

Twenty-one of the 31 FSA patients (68%) were able to be contacted by telephone to talk through their telehealth clinic experience. Not all questions were answered by every patient.

Results were:

Question 1 – Were you aware this appointment was a virtual/telehealth appointment? – 8 patients stated they were not aware, 13 were aware.
Question 2 – Were you happy with this arrangement pre-appointment? – 18 were happy, 2 were not, 1 did not answer.
Question 3 – Were you well prepared at the beginning of the appointment by the nurse? – 15 said yes – 3 said no – 3 no answer.
Question 4 – How would you rate the quality of the audio & visual of the telehealth appointment? – 6 poor sound – 15 satisfactory.
Question 5 – Were you able to understand what you were being told by the consultant? −17 – yes, 2 – no – poor sound, 2 no answer.
Question 6 – Do you rate this as a similar quality meeting with your consultant as an in-person appointment? - 18 – yes, 2 no, 1 don’t know.
Question 7 – Did the nurse have to explain anything after the appointment? – 14 – no, 6 – yes, 1 no comment.
Question 8 – Would you be happy to have a telehealth appointment again? (or prefer an in-person next time?) 19 – yes, 2 in person.
Question 9 – Any suggestions on how we can improve this new way meeting with patients? − 4 patients commented on improving sound quality, 1 if it could have been by telephone, and 1 felt improved awareness of what clinic to expect.
Question 10 – Overall level of satisfaction from the clinic – out of 10 (1 being very unsatisfied, 10 being very satisfied) a score of between 7 and 10 being scored by 18 (86%) of the 21 patients asked – a score of 10 out of 10 being the highest score of 1–10 (Table 2).
Question 11 – Would you prefer to see the neurosurgeon in-person next time, or happy with the virtual clinic set-up? 15 happy with virtual, 1 patient would have preferred a telephone call, 1 would prefer in person.

**Table 2.** Overall Level of Satisfaction.

Satisfaction Level	Number of Patients who Rated Satisfaction at this Level
Score of 10 – extremely satisfied	3
Score of 9	3
Score of 8	8
Score of 7	4
Score of 6	1
Score of 5	1
Score of 1 to 4	0
Did not/could not answer	1

Level of satisfaction out of a score of 10 – with 10 being extremely satisfied, 1 being not satisfied at all.

Other relevant comments were made as in Table 3.

**Table 3.** Other Relevant Comments.

Category of Comment	Comment from Patient
Quality of service	Was great having a nurse to help and able to summarise what the doctor said. The doctor was clearer than I had expected. Surgeon was able to show & explain my scan on a second screen. The sound was an problem. I could not hear the surgeon well (a problem conveyed by 4 patients). I could have been told by phone the outcome of my scan. That would save me & the health service time
Travel	I could not tolerate the drive (to hospital ‘B’) I would have cancelled appointment. This clinic saved a long drive. Happy to travel (to hospital ‘B’) I want the right doctor to see me. If I must travel, then that is ok. was wet on roads so good it was close to home. If it saves the doctor coming down to say what he had to say then a good idea. Desperate to see the specialist - would have driven if had to. Not travelling long distances is a help for the elderly. We don’t all have help to call upon to drive us.
Parking	No parking issues at (Hospital ‘C’) No travel & parking issues I have had to visit (Hospital ‘B’) for numerous Ophthalmology appointments. It's a nightmare when its wet & cold on the journey & finding parking.

### Patient Reduction (Savings) in Mileage

In total, there was a saving of 10,785 km for all FSA patients who attended the telehealth clinic. This was calculated using the travel distance from their home to Hospital ‘B’, where traditionally the clinics would have been held. The savings per patient who attended the telehealth clinic was estimated at NZ$61.14 (US$38) using an average car type −(50% mileage in a 1600cc petrol engine and 50% in a 2000cc diesel engine).

What has not been included is terms of monetary savings are parking costs at Hospital ‘B’, any overnight stay required, wear and tear on cars and time off work for the person who drove the patient to the appointment for an in-person appointment.

### Patient Reduction (Savings) in Time

Savings were calculated in the travel time taken for the patient to be driven to hospital ‘B’ for an in-person appointment, minus the time taken to drive to the actual rural hospital site of the telehealth clinic appointment. The total time saved for the 31 patients was 137 h. This did not include the time for any other person accompanying the patient. Given the age and frailty of many neurosurgery patients, it is likely that at least one other person would have accompanied the patient, therefore doubling the savings in time. Thus, the indirect costs and savings have not be assessed.

### Patients who did not Attend (DNA) Clinic

There were a total of four patients who did not attend the arranged telehealth clinic. This was a rate of 5.6%, calculated using the total number of clinic slots – 71 minus those who DNA the appointment – 4. Reasons for not attending the clinic were one patient residing in a nursing home had norovirus so unsafe to travel. One patient residing in a nursing home could not be organised on time for an ambulance to take the patient. Two patients did not know they had appointments made despite a phone call reminder to them the day before the appointment date.

### Patient Outcomes

All patients attending clinic have an outcome attached to their visit. These range from being discharged from the service to being added to the surgical waiting list. The highest number of outcomes is those patients discharged from the Neurosurgery service. This accounted for 12 patients (39%).

## Discussion

Question 1 reported that 8/21 (38%) of patients were not aware that they were attending a telehealth clinic. This suggests that repeated contact prior to the appointment maybe helpful as patients coming for a FSA in a hospital setting may assume the specialist will be there in person, despite this being communicated previously. The responses to question 2 and 3 overlapped with those to question 1.

Question 4 reported that the sound quality was an issue early in the schedule of clinics. This was resolved after the fourth clinic by the use of a dedicated laptop. Adaptability of the clinic process such as in this case improving the sound quality is one factor reported to influence the success and sustainability of telehealth clinics.^8^ Despite sound problems question 5 and 6 reported satisfactory results to being able to understand the discussion with the consultant and feeling the appointment was similar to an in-person consultation.

Question 7 reported that 6/21 patients found that the debrief after the appointment was needed, justifying the role of the nurse navigator attending the appointment.

Qualitative data and extracts from conversations above would suggest a high level of satisfaction from patients from their telehealth clinic appointment. High satisfaction was recorded for 18 of 21 (86%) patients asked and that 19 of 21 (90%) would have another telehealth clinic appointment in the future.

Satisfaction levels were also high with 18 respondents scoring a level of between 7 and 10 being 86%. This compares favourably with other studies of satisfaction with telehealth clinics.^9,10^

The low non-attendance rate of 4 out 71 (5.6%) potential clinic slots for new and follow-up patients was achieved due to the patient contact by the nurse navigators and administration staff. The improvement in non-attendance rates by telehealth use has been also been reported in other specialities^11^

### Evaluation of Telehealth Clinics

The introduction of any new service requires evaluation of its effectiveness to be accountable and provide a framework for further development. The National Quality Forum of the United States Department of Health and Human Services reported four domains for telehealth measurement including access to care, financial impact/cost, experience and effectiveness. Subdomains describe further aspects of the measurement framework.^12^

The aim of this study was to evaluate the role of the nurse navigator support for patients during telehealth clinics and the study used a structured questionnaire to evaluate the perceptions of access to care for patients (patient satisfaction survey questions 1, 2, 3 and 5) and the patient experience and satisfaction.(questions 6, 8, 9, 10 and 11) The effectiveness of the clinics was evaluated by the patients evaluation of the technical effectiveness especially regarding the sound quality of the link.(questions 4, 6 and 7), and the patient attendance rate. The financial impact and cost was evaluated by the evaluation of savings in mileage and patient time.

The questionnaire used for the patient satisfaction survey was developed specifically for the study rather than using pre-existing surveys.^13-17^ The reason for the development of a bespoke survey was to evaluate the role of the nurse navigator support for patients during the telehealth clinic process, rather than the telehealth clinic itself. Thus questions 3 and 7 specifically addressed this and to a lesser extent question 1. The questionnaire used has not as yet been validated and as discussed will evolve further. The use of non-validated questionnaires has been reported as the more common method of assessing telemedicine clinic being reported as used utilised in 77.5% and 76.9% of studies in metaanalyses.^10,13^

The development of a validated questionnaire as to the impact of a nurse navigator for the management of telehealth clinics has not yet been reported.

### Role of the Neurosurgical Nurse Navigator

Once a patient has been referred to a clinic, there can be a delay before being seen with a number of barriers in any healthcare system before secondary or tertiary care can be given. A ‘lay navigator’ or patient pathway coordinator can help with navigating the administrative side of this, though this role is usually undertaken by a trained non-clinical administrator or volunteer. A nurse navigator is a term that can be applied to a professionally registered nurse who, as well as providing care to patients, can also help the patient navigate the system and access resources before and after the clinic. It can also be helpful for the nurse navigator to be present during the clinic to provide psychosocial support. This role has been developed in delivery of care for patients with chronic diseases and in oncological practice.^18^

The role of the neurosurgical nurse navigators was in setting up the clinics, identifying the patients, contacting the patients prior to the clinic, connecting with the telehealth platform, being in the clinic with the patient, debriefing the patient after the clinic when needed and acting on further outcomes such as organising scans and follow-up appointments (Table 4). Although the nurse navigator was present in clinic with the patient, they were only needed in two instances for help with neurological examination: in one case to assess upper limb strength and the other to help with gait analysis. The role of a person in clinic to help with neurological examination has been reported in a number of studies.^19-21^ For these clinics pre-clinic screening meant that those needing a face-to-face consultation were not offered a telehealth appointment and that most patients did not need a physical examination in person to guide future management.^20^ The role of the nurse navigator involved in the patients pathway in the pre-clinic and post-clinic phases was felt to be beneficial to both patient and specialist physician. This is different to having a separate local nurse assistant in the clinic with the patient. The impact of this was not assessed but local nurse assistant support by a neurosurgical nurse navigator may be an area for future development.

**Table 4.** Role of the Neurosurgical Nurse Navigator in the Telehealth Clinics.

Pre-clinic	Early: Identification of patients on waiting list Screening of patients suitable for telehealth Liaising with specialist neurosurgeon Organising pre-clinic imaging where needed Organising availability of imaging on tertiary hospital server Prior to clinic: Confirming clinic appointment with patient including where to go and type of clinic Confirming IT set up in clinic
During clinic	Making IT connection with specialist prior to patient arrival and optimising audio-visual set up with local IT support Bringing patient to screen and showing patient how IT works Assisting in examination if needed
Post-clinic	Immediate: Debrief with patient once appointment finished – allows for reconnection with specialist if any points need clarifying Implementation of management plan – eg, further investigations or appointments. Late: Checking on booked imaging and follow-up appointments

### Telehealth Clinic Models

There are a number of different ways of delivering telehealth clinics.^6,8,20^ These telehealth clinics may be telephone only or videoconferencing from an office base or hospital with the patient at home or at a local hospital.

In many cases, the attendance of patients at a local hospital facility and then communicating with the tertiary centre is the easiest way to gain access to a specialist opinion and is closest to the in-person clinic format. This model can be particularly useful where there maybe issues with access or use of technology, where patients do not have the resources.^1^ This model was used in 68% of telehealth services used in rural Australia.^8^

Other models include direct telehealth consultations with the patient at home. This requires technological access, resources and capability which may be more difficult for some patient groups.^1^ In some circumstances, there is a financial driver from the healthcare provider to promote telemedicine encounters especially where it perceived that there may be cost savings for clinical overheads, not needing support or nursing staff, not needing clinic rooms and improved clinic workflow with potential reduction in allotted time needed.^6^

A hybrid model of the nurse navigator fulfilling the roles as described, but the telehealth clinic being held in the patients home, with the specialist physician and the nurse navigator also dialling in as a three-way consultation is being explored. The challenges to implementation will include technological, eg, speed of internet connection, scheduling eg, time taken between appointments, and suitability of patients.

For some patients, the in-person encounter and being able to meet in person, at an early stage, the surgeon treating them who may also be operating on them in the future, is important.^1^ Where available patients should be given the option to choose which clinic model suits them.

Clinics held in local hospitals have always been welcomed by patients and their families.^9^

### Limitations

There was a response rate of 68% for this study which may introduce a response bias in the survey. This study was a single site study of triaged long-waiting neurosurgical outpatients using a single model of telehealth delivery. While the study provides valuable insights into the initial feasibility of telehealth clinic supported by neurosurgical nurse navigators, the limited sample size, potential selection bias, response bias and lack of long-term follow-up pose challenges to the generalizability of the results. Enhancements in study design, including a larger and more diverse sample, a control group and addressing technology-related issues are areas for future development.

## Conclusions

Patients had a high satisfaction rate for these telehealth clinics. Patients cited the convenience of reduced travel, ease of parking and reduced personal costs without any reduction in the quality of the consultation with the specialist.

The role of the nurse navigators at every stage of the clinic process was important in the success of the clinics. Contact with patients before, during and after the clinic contributed to high satisfaction rates and low non-attendance rates. As these are the first set of scheduled clinics managed in this way, there are opportunities to improve the experience for patients.
